# The dynamic features of long-term habituation of negative emotions: evidence from eye-tracking and hypothalamic-pituitary-adrenal axis research

**DOI:** 10.3389/fpsyt.2026.1845849

**Published:** 2026-10-07

**Authors:** Fuqiang Wang, Shichang Yang, Liang Li, Lubin Wang, Shuai Chen, Jiakai Chang, Qun Yang, Kai Gao, Pinhong Chen

**Affiliations:** 1 Beijing Institute of Basic Medical Sciences, Beijing, China; 2 Department of Medical Psychology, the Second Affiliated Hospital of Naval Medical University (Shanghai Changzheng Hospital), Shanghai, China; 3 The Second Affiliated Hospital of Henan Medical University, Xinxiang, China; 4 Clinical Medical Center of Traditional Chinese and Western Medicine for Psychiatric Diseases of Henan Medical University, Xinxiang, China

**Keywords:** eye-tracking, hypothalamic–pituitary–adrenal axis, negative emotional habituation, resilience, trait anxiety

## Abstract

**Objectives:**

Negative emotional habituation (NEH) plays a crucial role in individuals’ survival and adaptation. However, systematic research on long-term negative emotional habituation remains limited. This study aimed to explore the dynamics of the habituation process during prolonged repeated exposure to negative stimuli, from the perspectives of eye-tracking and hypothalamic–pituitary–adrenal (HPA) axis responses.

**Methods:**

Employing a 2 × 7 mixed factorial design, we enrolled 50 healthy male undergraduates and randomly assigned them to moderate- or high-intensity negative-image groups. Negative stimulus intensity served as the between-subject factor, and repeated exposure across seven consecutive daily testing sessions served as the within-subject factor. A single-blind procedure was adopted. Baseline trait anxiety and resilience were assessed. On each testing day, we concurrently obtained subjective ratings (valence, arousal), eye-tracking indices [mean fixation duration (MFD), pupil size], and salivary cortisol samples.

**Results:**

Over the seven-day repetition, valence increased over time while arousal decreased. MFD increased significantly while pupil size decreased significantly, with both indices differing by stimulus intensity. Daily changes in salivary cortisol exhibited a significant downward trend, with a steeper decline under high-intensity negative stimulation. Cross-system coupling varied by intensity: under moderate intensity, valence and cortisol habituation were positively correlated, whereas under high intensity, this association was not significant. Additionally, trait anxiety was significantly negatively correlated with pupil habituation, and resilience moderated the relationship between trait anxiety and valence habituation at a marginally significant level.

**Conclusion:**

These findings indicate that NEH is not a unitary pathway but a dynamic process jointly realized by multiple systems with distinct sensitivities and convergence rhythms. The results reveal nonlinear cross-level coupling in NEH and may inform methodological bases for timing, dose optimization, and process evaluation in exposure-based therapies.

## 1 Introduction

Negative emotional habituation (NEH) refers to an adaptive process in which subjective experience and physiological responses progressively diminish and stabilize over time when individuals are repeatedly exposed to negative emotional stimuli (1–3). This mechanism is regarded as one of the fundamental processes for maintaining emotional homeostasis and reducing stress load. Although traditionally conceptualized as a unitary mechanism, whether subjective, attentional, and neuroendocrine systems follow synchronized habituation trajectories remains a key unresolved theoretical question. In the psychological treatment of post-traumatic stress disorder (PTSD), habituation constitutes a key component through which exposure-based therapies (e.g., prolonged or imaginal exposure) achieve their efficacy (4). Conversely, aberrant habituation is typically associated with persistent hypervigilance, intensified negative affect, and elevated physiological arousal (5). This suggests that habituation serves as a “bridge” for understanding how basic emotional processing relates to the maintenance and alleviation of clinical symptoms.

Numerous studies have shown that an attentional bias (AB) toward threat-relevant stimuli constitutes a common feature of disorders including PTSD and anxiety disorders (4, 6, 7). Attention is shaped by the emotional significance of stimuli, personal experience, and evolutionary demands, yielding automatic and prioritized processing for cues related to survival, safety, or core needs (7). Owing to its high temporal resolution and capacity to directly and precisely quantify attentional orienting, maintenance, and shifting, eye-tracking has been widely employed in AB research (8, 9). In a sample of healthy undergraduates, higher fear evoked by picture stimulus was associated with shorter mean fixation duration (MFD). With repeated exposure, participants’ fear levels decreased over time, but their MFD increased correspondingly (8). The results indicate that higher fear during exposure may induce attentional avoidance. Moreover, a meta-analysis by Armstrong et al. (10) reported that among individuals with anxiety- and fear-related disorders, those with high anxiety exhibited significantly shorter gaze durations to fearful stimuli than to other stimuli, consistent with active avoidance. In contrast, individuals with low anxiety showed no difference in fixation time between fear-related and other stimuli. According to the vigilance–avoidance model (7, 11), individuals with anxiety- and fear-related symptoms exhibit emotional and physiological arousal when confronted with threat. Individuals then tend to engage in avoidance strategies to reduce the unpleasant emotional state. Although negative affect may be relieved in the short term, the underlying anxiety-related symptoms may persist or even worsen.

The long-term impact of negative emotional stimuli on the stress system is a central link for understanding the cognitive–physiological mechanisms of emotional disorders, and the HPA axis response has become a key biomarker in affective neuroscience research (12, 13). Existing studies indicate that repeated exposure to psychological stressors leads to a decreasing trend in cortisol responses, a physiological change referred to as habituation of the HPA axis (12). A systematic review reported that early cortisol overaccumulation modifies activity within critical brain regions, consequently impacting habituation of the HPA axis (14). Findings by Manigault et al. corroborate this conclusion: in repeated-stress paradigms, individuals with high initial stress reactivity were more likely to exhibit “habituation” (i.e., progressive decreases in HPA-axis response with additional exposures), whereas those with lower initial reactivity were more prone to “sensitization” (i.e., progressive increases in HPA-axis response) (13). Moreover, individual differences frequently influence habituation of the HPA axis. Researchers have found that resilience is a predictor of cortisol habituation. Individuals with greater resilience exhibit lower overall cortisol output during stress and appear more prone to habituate across repeated stressors (12). Furthermore, Yang et al. (15) measured the cortisol awakening response (CAR) over multiple days in college students and found a significant positive association between CAR variability and trait anxiety. Trait anxiety is a stable personality characteristic that reflects persistent and pervasive stress responses to potential threats (16). Accordingly, these findings suggest that individuals with high trait anxiety may have instability in baseline HPA-axis regulation, making it more difficult to achieve stable hormonal control via HPA-axis habituation under repeated negative stimulation.

In addition, exposure interval and repetition count constitute critical temporal factors shaping NEH. Previous studies have largely focused on within-session short repeats or multi-day, spaced-repetition designs. For example, Liu et al. asked participants to undergo two repeated exposures to stressors within a single session to probe neural habituation under acute stress (17). Szeska and co-workers had spider-phobic participants complete three sequential standardized tarantula exposures to test how transcutaneous auricular vagus nerve stimulation affects psychophysiology during extinction (18). Moreover, animal studies have used a once-every-three-days schedule to repeatedly re-expose PTSD model mice to the same traumatic context to assess the impact of traumatic memory on fear extinction (19). However, within-session designs carry arousal carryover confounds and only capture transient sensory adaptation, while spaced multi-day designs introduce circadian and daily stress variability that disrupts continuous adaptation trajectory mapping (20). We adopted a between-subject intensity design to avoid order and carryover effects, and our consecutive-days fixed-time paradigm addresses these limitations by controlling circadian confounds, aligning with clinical exposure dosing, and capturing full nonlinear habituation trajectories. To date no study in healthy populations has employed a “consecutive-days, fixed-time” exposure paradigm to systematically map the multi-day habituation trajectories of subjective experience, attentional processing, and HPA-axis responses.

Here, we employ a seven-day continuous repeated-exposure paradigm with two negative-intensity conditions (moderate and high). To establish robust baseline habituation dynamics with minimal endocrine cycle confounds in this initial investigation, we recruited an all-male sample and controlled diurnal hormonal variation via fixed daily testing. On each experimental day, we concurrently collect subjective ratings (valence and arousal), eye-tracking indices (MFD and pupil size), and salivary cortisol to characterize individual trajectories of emotional habituation. In parallel, we assess trait anxiety and psychological resilience to examine how individual differences modulate this process. From a diathesis–stress perspective (21–23), multi-system habituation outcomes reflect the interplay of vulnerability and protective factors: trait anxiety acts as a stable vulnerability marker, while resilience may buffer anxiety-related impairment in adaptation. We therefore planned moderation analyses to test whether resilience buffers the association between trait anxiety and NEH trajectories.

Specifically, we hypothesized that long-term NEH is not unitary: across seven days of exposure, valence and mean fixation duration would increase, while arousal, pupil size, and salivary cortisol responses would decrease. We further hypothesized that resilience would moderate the relationship between trait anxiety and habituation outcomes across systems. With this design, we can evaluate synchrony and divergence among subjective, attentional, and HPA responses over multi-day exposure, elucidate the effects of intensity and individual differences on NEH courses, and, in turn, provide clinically usable evidence for individualized exposure interventions, outcome evaluation, and monitoring relapse risk.

## 2 Methods

### 2.1 Participants

Participants were recruited through advertisements posted on social media. Experimental data were collected between January and May 2024. All participants were enrolled at a university in Beijing, China (*M_Age_=*26.3, *SD_Age_* = 2.9, *Range* = 22-35), and were pursuing master’s degrees. It is important to acknowledge that endogenous endocrine variability is not exclusive to women; both sexes exhibit dynamic hormonal fluctuations, but with distinct temporal profiles and magnitudes. In men, gonadal and adrenal hormone fluctuations are primarily diurnal and ultradian (pulsatile) in nature (13, 24), which were tightly controlled in this study via fixed daily testing times and standardized pre-sampling procedures. In contrast, the female menstrual cycle introduces large-amplitude, multi-day endocrine shifts that would directly overlap with our 7-day exposure window and cannot be fully controlled via procedural standardization alone (25, 26). The exclusion criteria were: 1) a history of psychiatric disorders; 2) physical illnesses affecting emotional or physiological indicators; 3) uncorrected visual impairments; 4) inability to participate at the same time period for seven consecutive days; 5) use of any medication during the study. This study was approved by the Beijing Institute of Basic Medical Sciences Ethics Committee(AF/SC-08/02.482), and written informed consent was obtained from all participants.

Ultimately, 52 participants were recruited for this study. However, during the experiment, 2 participants dropped out midway, leaving 50 participants in the final sample. Prior to the formal experiment, they were randomly assigned into two groups: the moderate-intensity negative group and the high-intensity negative group, with 25 participants in each.

### 2.2 Mental health questionnaires

The Trait Anxiety Inventory (TAI) is used to assess individuals’ habitual tendency to experience anxiety in response to potential threats or stressful situations at a long-term and stable personality-trait level, which distinguishes it from “state anxiety” that is specific to particular situations (16). In this study, the Chinese version of the TAI (27) was used. In the present study, Cronbach’s alpha for this questionnaire was 0.930.

The Resilience Scale for Chinese Adults (RSCA) is designed to measure the stable personality trait that enables individuals to recover from psychological trauma and return to their previous level of functioning after experiencing major stress events (28). In the present study, the Cronbach’s alpha for this questionnaire was 0.913.

### 2.3 Experimental materials

This study adopted images from the International Affective Picture System (IAPS) as experimental materials (29). First, images with a valence score below 4.0 were selected from the IAPS. Next, based on the original valence, arousal, and dominance ratings of each picture, clustering was performed using the self-organizing map (SOM) neural network method (30), resulting in four levels of negative emotional images (mild, moderate, high, and extreme negative), ordered by increasing emotional intensity. Finally, two intensity levels of negative emotional images were chosen as experimental materials: moderate-intensity (valence = 2.817 ± 0.547, arousal = 5.393 ± 0.317, dominance = 4.042 ± 0.344) and high-intensity (valence = 2.647 ± 0.904, arousal = 6.054 ± 0.332, dominance = 3.592 ± 0.545), with 80 images in each category. The moderate-intensity level corresponded to the second cluster, and the high-intensity level corresponded to the third cluster. This selection was made to cover ecologically valid stress intensity ranges while avoiding extreme stimuli that might cause undue distress and ethical risks.

### 2.4 Acquisition and analysis of eye-tracking data

This study employed the EyeLink 1000 Plus Desktop Mount eye-tracking system (SR Research Ltd., Mississauga, Canada) (31) to record monocular (right eye) gaze data at a sampling rate of 2000 Hz. The experiment was conducted in a light-shielded and soundproof behavioral laboratory, with participants seated at a fixed distance of 65 cm from a Dell monitor (19 inches, 1440 × 900 px resolution, 75 Hz refresh rate). Before the experiment, a 9-point calibration (error ≤ 0.5° of visual angle) and validation were performed. During the experiment, participants’ heads were stabilized with a chin rest and forehead support. Stimulus presentation and event marking were implemented using Experiment Builder v2.3.38 (32). Eye movements were analyzed with EyeLink Data Viewer, with the entire image defined as the area of interest (AOI) (33). Based on the research objectives, the MFD (total fixation time per picture divided by the number of fixations) and pupil diameter were extracted from the raw data as key eye-tracking indices. Since the raw data were not normally distributed, they did not meet the assumptions required for ANOVA. To facilitate further statistical analysis, a square root transformation was applied, followed by a normality test to ensure the data met the assumption of normal distribution (15, 34). Extreme values were defined as data points exceeding three standard deviations from the mean and were replaced using the corresponding upper and lower 5% percentiles. A total of 1.82% of MFD values and 1.57% of pupil size values were identified as outliers and winsorized. Sensitivity analyses confirmed that core results remained consistent without winsorization.

### 2.5 Acquisition and analysis of salivary cortisol

On each collection day, participants were required to provide two saliva samples, one before the start of the daily experiment and one after the experiment ended. In addition, within 30 minutes prior to each sampling, participants were instructed to refrain from eating, drinking beverages (including those containing alcohol, caffeine, or juice), brushing teeth, rinsing, or smoking. Saliva samples were collected using a specialized sampling device (Salivette, SARSTEDT, Germany) (35). After collection, all saliva samples were stored at -80°C until analysis. Before analysis, the saliva samples were thawed naturally in a 4°C refrigerator. The samples were then centrifuged (1000×g, 4°C, 2 min), and the supernatant was collected for subsequent analysis (35).

Salivary cortisol was measured using enzyme-linked immunosorbent assay (ELISA, USCN LIFE SCIENCE INC, China) to assess cortisol concentrations before and after the experiment. Before analysis, cortisol data were cleaned and processed for outliers to meet the assumptions of statistical analyses. As cortisol data typically exhibit a positively skewed distribution, a natural logarithmic transformation was applied, followed by normality testing to ensure the data met the assumption of normal distribution (15, 34). Outliers were defined as data points exceeding three standard deviations from the mean and were replaced with the corresponding upper and lower 5% percentiles (36). Approximately 2.14% of cortisol data points were winsorized. This procedure was chosen over exclusion to preserve the continuity of longitudinal trajectories, and sensitivity analyses verified the robustness of core findings.

### 2.6 Procedure

On the first day of arrival at the laboratory, the experimenter met with the participants to explain the experimental procedure and precautions, and asked them to complete demographic information as well as the TAI and RSCA questionnaires, which were administered only at baseline (Day 1) as they measure stable trait-level characteristics. Subsequently, the first saliva sample of the experiment was collected. Next, the eye-tracking device was adjusted and calibrated for each participant. Before the formal experiment began, three neutral practice images were presented to the participants, each displayed for 6 seconds. Participants were asked to rate the valence and arousal of the images using a 9-point Likert scale. After each evaluation and before the next image was presented, a fixation cross appeared at the center of the screen for 3 seconds as an interstimulus interval. After the practice session, the formal experiment began. In this stage, participants were presented with 80 negative emotional images of either moderate or high intensity, depending on their group assignment. The presentation duration and rating method were identical to those in the practice phase. Immediately after the experiment, another saliva sample was collected from each participant. The entire experiment lasted approximately 16 minutes (**Figure 1**). Each participant repeated the experiment daily at the same time for seven consecutive days. The same set of 80 negative images was presented across all seven days, with pseudo-randomized presentation order for each daily session to control for sequence-specific memory effects. Participants were not informed of the repeated image set prior to the experiment to avoid anticipatory response biases, and a full debriefing was provided after the final testing session.

**Figure 1:**
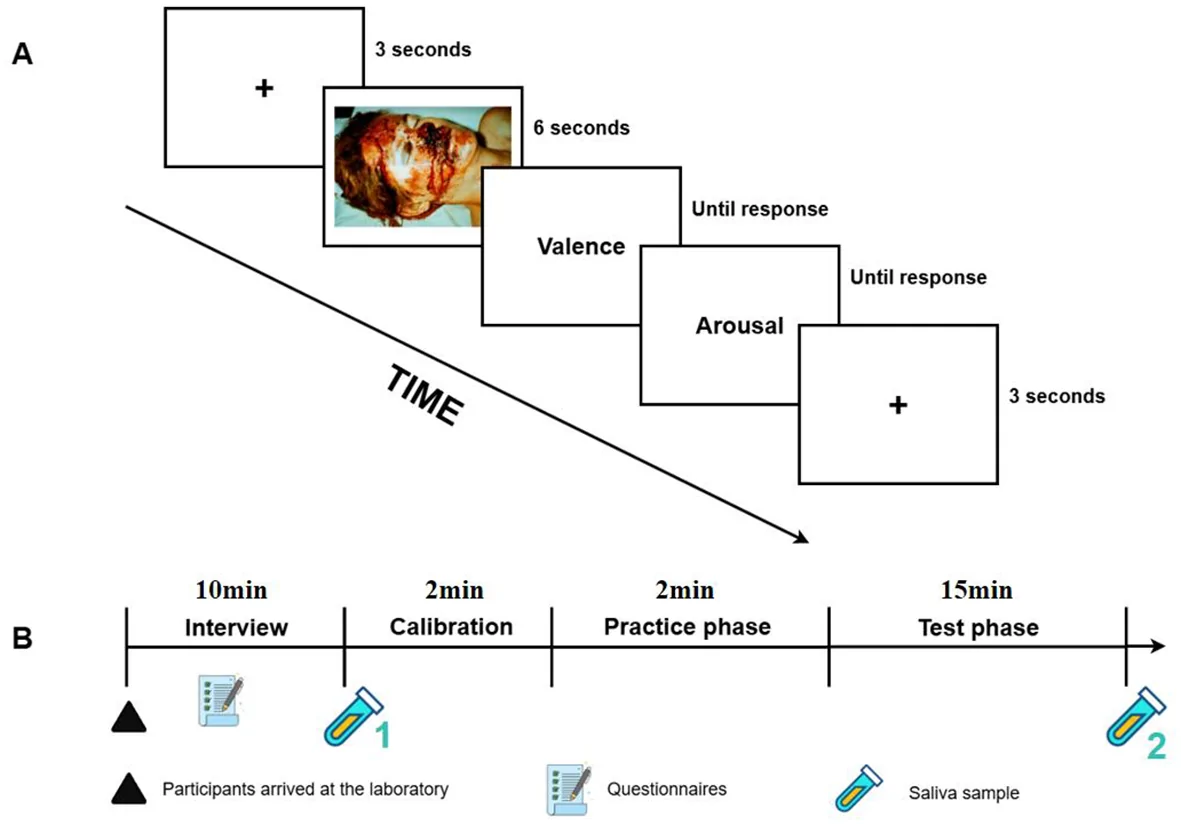
Experimental procedure. **(A)** Timeline of a single trial. Each trial started with a 3-second fixation cross, followed by a 6-second presentation of a negative image. Participants then completed sequential valence and arousal ratings in a self-paced manner (until response), and the trial ended with a 3-second inter-trial fixation cross. **(B)** Timeline of each daily testing session. Saliva sample 1 was collected immediately before eye-tracker calibration, and saliva sample 2 was collected immediately after the end of the test phase, with an approximate 20-minute interval between the two samples. Each daily session consisted of a 10-minute interview/check-in period, 2-minute eye-tracker calibration, 2-minute practice phase, and 15-minute formal picture-viewing test phase. Demographic and trait questionnaires were only administered during the interview period on Day 1; the procedure was identical across Days 2–7 with no additional questionnaires. The total duration of each daily session was approximately 30 minutes.

### 2.7 Statistical analysis

All data were statistically analyzed using SPSS version 27.0.1. Prior to repeated-measures ANOVA, normality of model residuals and homogeneity of variance were formally tested to verify that parametric test assumptions were met. Raw data distributions were inspected earlier to guide appropriate data transformation procedures. When ANOVA results were significant, *post hoc* multiple comparisons were conducted using the least significant difference method. Specifically, time (seven consecutive days of repeated experiments) and negative intensity (moderate- and high-intensity) were treated as independent variables. A 2×7 mixed-design repeated-measures ANOVA was conducted to examine daily differences in subjective ratings (valence, arousal), eye-tracking indices (pupil size, MFD), and daily fluctuations in salivary cortisol.

The overall variance of subjective ratings, eye-tracking indices, and cortisol changes across the seven days was used as a habituation index. This index was chosen because it captures the full magnitude of adaptive change across the entire time course, without assuming a strictly linear trajectory, which is consistent with prior longitudinal habituation research (15, 34). Although variance is not inherently direction-sensitive, repeated-measures ANOVAs confirmed significant main effects of time with consistent directional trajectories for all indicators. Thus, cross-day variance in the present dataset predominantly reflects systematic adaptive change rather than random fluctuation. Independent-samples t-tests were then conducted with negative intensity (moderate vs. high) as the independent variable to assess the effect of intensity on habituation.

Finally, correlation analysis and hierarchical linear regression were performed to investigate whether trait anxiety and psychological resilience influence NEH under different negative intensity levels. Three models were built sequentially: Model 1 included age as a covariate; Model 2 added negative intensity, trait anxiety, and resilience as main predictors; Model 3 added the trait anxiety × resilience interaction term.

## 3 Results

Independent-samples t-tests confirmed no significant between-group differences in all pre-exposure baseline variables: age (t (48) = 0.097, p = 0.923), trait anxiety scores (t (48) = -0.013, p = 0.989), resilience scores (t (48) = 0.669, p = 0.507), and Day 1 pre-experiment salivary cortisol (t (48) = -0.289, p = 0.774). The two groups were well matched following random assignments.

### 3.1 Interday differences in subjective ratings

We first conducted descriptive statistics of the subjective ratings of valence and arousal across seven consecutive days for the two participant groups receiving negative stimuli of differing intensity. (see **Supplementary Table 1** in the **Supplementary Material**). Consistent with the significant time main effects reported below, valence ratings showed a gradual increase over days, with the moderate-intensity group exhibiting higher daily valence than the high-intensity group; in contrast, arousal ratings declined over time, and daily arousal in the moderate-intensity group was consistently lower than in the high-intensity group(see **Figure 2A**). Next, repeated-measures ANOVAs were performed on the valence and arousal ratings. For valence ratings, the main effect of time was significant (*F _(6, 288)_ =* 3.777, *p* < 0.001), while neither the main effect of negative intensity nor the time × negative intensity interaction was significant (negative intensity: *F _(1, 48)_ =* 0.713, *p* = 0.403; time × negative intensity: *F _(6, 288)_* = 1.705, *p* = 0.120). For arousal ratings, the main effect of time was significant (*F _(6, 288)_* = 5.474, *p* < 0.001), whereas the main effect of negative intensity and the time × negative intensity interaction were nonsignificant (negative intensity: *F _(1, 48)_* = 0.084, *p* = 0.773; time × negative intensity: *F _(6, 288)_* = 0.981, *p* = 0.438) (see **Supplementary Table 2** in the **Supplementary Material** for *post hoc* results).

**Figure 2:**
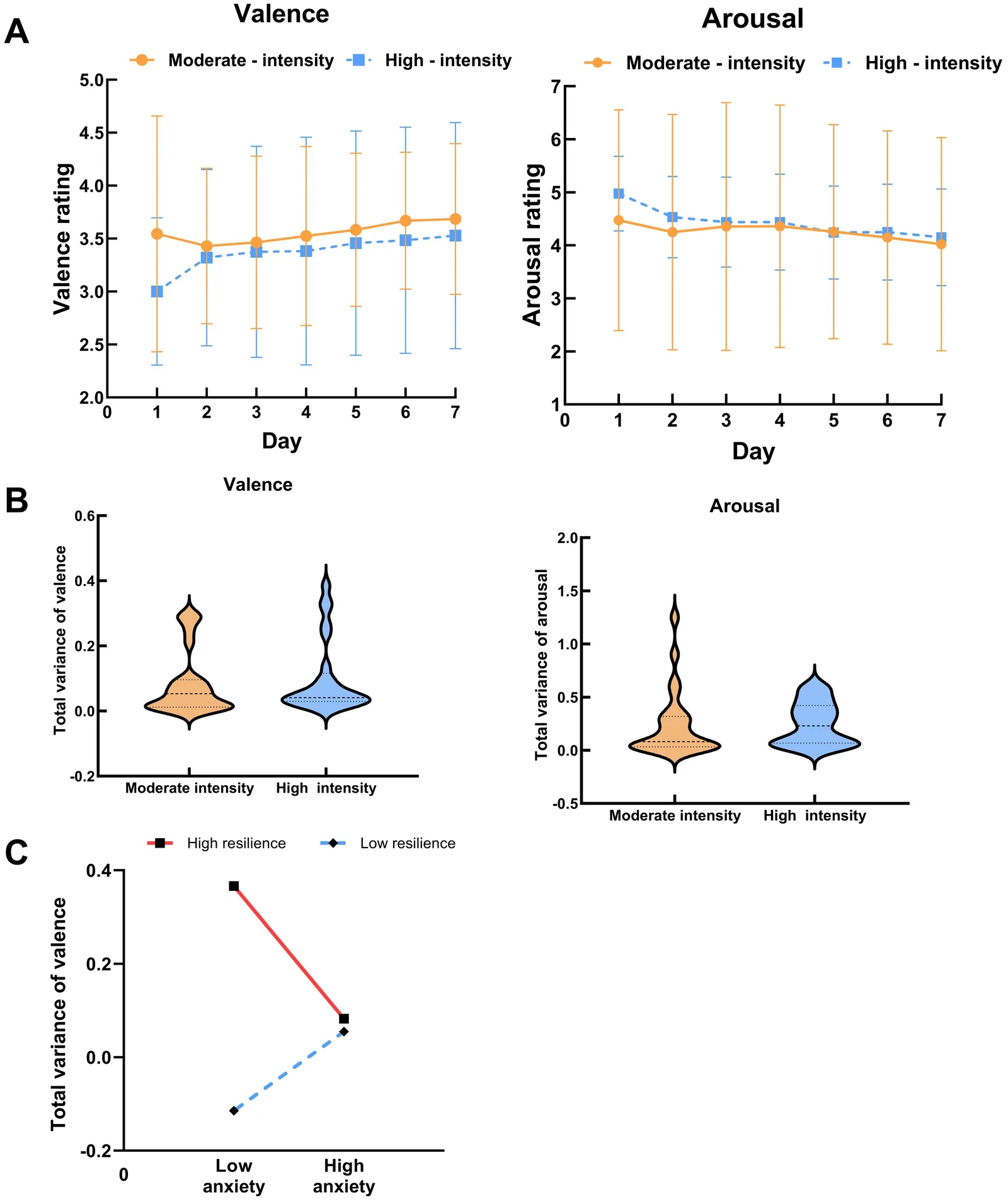
Tests of differences in subjective valence and arousal ratings. **(A)** Results of valence and arousal ratings across seven consecutive experimental days under moderate-intensity and high-intensity conditions; the left panel shows valence, and the right panel shows arousal. **(B)** Violin plots of data distribution under different negative intensity conditions, using the overall variance of ratings across seven days as habituation indices; the left panel represents valence, and the right panel represents arousal. **(C)** Moderating effect of resilience on the relationship between trait anxiety and overall valence variance.

Next, independent-samples t-tests were conducted on the habituation indices of valence and arousal. The results (see **Figure 2B**) showed that differences between the two negative intensity levels were not significant for either dimension (valence: *t _(48)_* = -0.517, *p* = 0.608, *Cohen’s d* = 0.106; arousal: *t _(48)_* = 0.449, *p* = 0.656, *Cohen’s d* = 0.718). We then conducted hierarchical regression analyses to examine the predictive effects of age, negative intensity, trait anxiety, resilience, and their interaction (trait anxiety × resilience) on the habituation indices of valence and arousal, sequentially building three models (see **Table 1**). The explanatory power of Model 3 for overall valence variance increased significantly (*R²* = 0.229, *F* = 2.609, *p* < 0.05). Trait anxiety × resilience showed a marginally significant predictive effect on overall valence variance (*β* = -0.105, *p* = 0.065), indicating a potential moderating effect of resilience on the relationship between trait anxiety and valence habituation. However, Model 3 did not significantly improve the explanatory power for overall arousal variance (*R²* = 0.170, *F* = 1.798, *p* = 0.133). Trait anxiety × resilience, along with age, negative intensity, trait anxiety, and resilience, did not significantly predict overall arousal variance.

**Table 1:** Hierarchical regression results of subjective ratings.

Variables	Total variance of valence			Total variance of arousal		
	Model 1	Model 2	Model 3	Model 1	Model 2	Model 3
Constant	0.302	-0.203	-0.401	1.275	0.539	0.300
Age	-0.005	-0.017	-0.008	-0.035	-0.052	-0.041
Negative intensity	/	-0.112	-0.088	/	-0.059	-0.031
Trait anxiety	/	-0.004	-0.004	/	-0.007	-0.007
Resilience	/	0.013	0.011	/	0.017	0.015
Trait anxiety × Resilience		/	-0.105	/	/	-0.127
R^2^	0.001	0.166	0.229	0.020	0.134	0.170
F	0.054	2.235	2.609*	0.993	1.744	1.798

“/” indicates that the variable was not included in the corresponding model. ∗*p* < 0.05. N=50.

To better understand the moderating effect of resilience, we subsequently conducted a simple slope analysis. As shown in **Figure 2C**, for participants with low resilience (M − 1SD), trait anxiety positively predicted overall valence variance, but the effect was relatively small (*simple slope* = 0.008, *t* = 0.946, *p* = 0.349). Among participants with high resilience (M + 1SD), trait anxiety showed a non-significant negative trend *(simple slope* = -0.136, *t* = -1.516, *p* = 0.136). Although neither simple slope reached statistical significance, the opposite directional pattern across the two groups was consistent with the hypothesized buffering effect of resilience on trait anxiety-related adaptation difficulties, providing tentative directional support for the marginal interaction.

### 3.2 Interday differences in eye-tracking indices

Descriptive statistics of MFD and pupil size across seven consecutive days were conducted for the two groups (see **Supplementary Table 1** in the **Supplementary Material**). Consistent with the significant time main effects reported below, MFD showed a gradual increase, with the high-intensity group exhibiting higher daily MFD than the moderate-intensity group; by contrast, pupil size decreased over time, and daily pupil size was consistently larger in the high-intensity group than in the moderate-intensity group (see **Figure 3A**). Next, repeated-measures ANOVAs were conducted on two eye-tracking indices. For MFD, the main effects of time and negative intensity were significant (time: *F _(6, 288)_* = 10.150, *p* < 0.001; negative intensity: *F _(1, 48)_* = 4.455, *p* < 0.05), whereas the time × negative intensity interaction was not significant (time × negative intensity: *F _(6, 288)_* = 0.760, *p* = 0.602). For pupil size, both the main effects of time and negative intensity were significant (time: *F _(6, 288)_* = 5.581, *p* < 0.001; negative intensity: *F _(1, 48)_* = 3.833, *p* < 0.05), whereas the time × negative intensity interaction was nonsignificant (time × negative intensity: *F _(6, 288)_* = 0.481, *p* = 0.822) (see **Supplementary Table 3** in the **Supplementary Material** for *post hoc* results).

**Figure 3:**
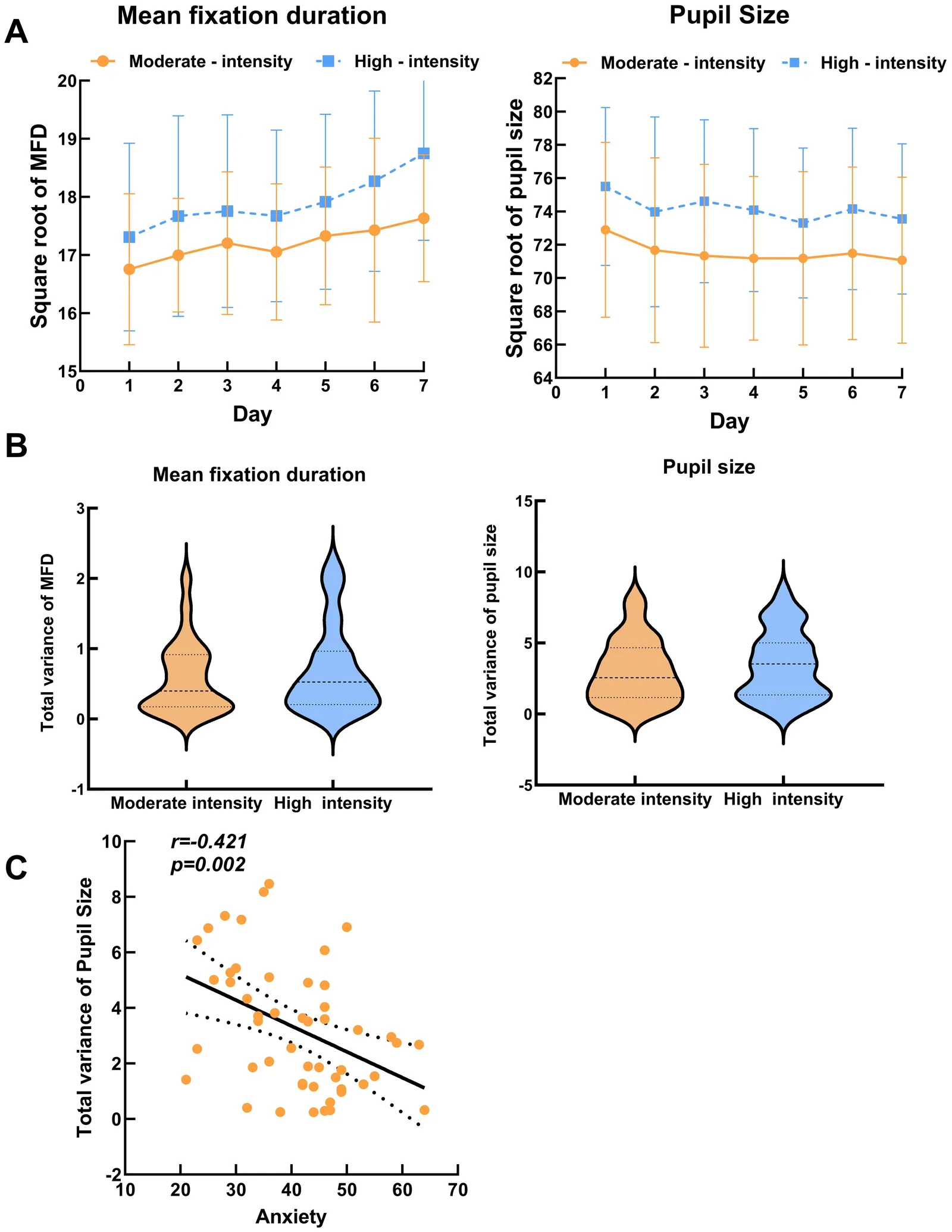
Tests of differences in eye-tracking indices. **(A)** Results of MFD and pupil size across seven consecutive experimental days under moderate-intensity and high-intensity conditions; the left panel shows MFD, the right panel shows pupil size. **(B)** Violin plots showing the data distribution under different negative intensity conditions, using the overall variance of eye-tracking indices across seven days as habituation indices; the left panel represents MFD, the right panel represents pupil size. **(C)** Scatterplot of total variance of pupil size and trait anxiety. MFD, Mean fixation duration.

Next, independent-samples t-tests were conducted on the habituation indices of MFD and pupil size. Results (see **Figure 3B**) showed that the differences between negative intensity conditions were nonsignificant for both eye-tracking measures (MFD: *t _(48)_* = -0.389, *p* = 0.699, *Cohen’s d* = -0.110; pupil size: *t _(48)_* = -0.665, *p* = 0.509, *Cohen’s d* = -0.188). We then conducted hierarchical regression analyses to examine the predictive effects of age, negative intensity, trait anxiety, resilience, and their interaction (trait anxiety × resilience) on the habituation indices of MFD and pupil size, building three models sequentially. The results indicated no significant effects for MFD. However, for pupil size, both Model 2 and Model 3 reached overall significance (Model 2: *F* = 2.860, *p* = 0.034; Model 3: *F* = 2.423, *p* = 0.050). Moreover, in both models, trait anxiety significantly predicted overall variance in pupil size (Model 2: *β* = -0.114, *p* = 0.006; Model 3: *β* = -0.113, *p* = 0.007) (see **Table 2**).

**Table 2:** Hierarchical regression results of pupil size.

Variables	Total variance of pupil size		
	Model 1	Model 2	Model 3
Constant	-0.069	9.928	9.456
Age	0.127	0.019	0.04
Negative intensity	/	0.372	0.428
Trait anxiety	/	-0.114**	-0.113**
Resilience	/	-0.034	-0.038
Trait anxiety × Resilience	/	/	-0.25
R^2^	0.025	0.203*	0.216
F	1.247	2.86*	2.423*

“/” indicates that the variable was not included in the corresponding model. *∗p* < 0.05,**p<0.01. N=50.

To further illustrate the association between trait anxiety and the pupil habituation index, a scatterplot of trait anxiety and total variance of pupil size was plotted (see **Figure 3C**). The results showed a significant moderate negative correlation between trait anxiety and pupil size (*r* = -0.421, *p* = 0.002). This finding corroborates the results of the regression analyses, further supporting the predictive role of trait anxiety in pupil responses during the process of NEH.

### 3.3 Interday differences in cortisol indices

Descriptive statistics of daily changes in salivary cortisol over seven consecutive days for the two groups are shown in **Supplementary Table 1** in the **Supplementary Material**. Consistent with the significant time main effects reported below, the day-to-day change of salivary cortisol showed a declining trend over the 7 days; specifically, the high-intensity group exhibited greater cortisol change than the moderate-intensity group on the first two assessment days, the moderate-intensity group showed greater change on days 3–5, and no between-group differences were observed on the final two days (see **Figure 4A**). Next, repeated-measures ANOVAs were conducted on daily salivary cortisol changes. The main effect of time was marginally significant (*F _(6, 288)_* = 2.048, *p* = 0.059), whereas the main effect of negative intensity and the time × negative intensity interaction were not significant (negative intensity: *F _(1, 48)_* = 0.418, *p* = 0.521; time × negative intensity: *F _(6, 288)_* = 0.783, *p* = 0.584) (see **Supplementary Table 4** in the **Supplementary Material** for *post hoc* results). We further conducted independent samples t-tests on the habituation indices of salivary cortisol. Results (see **Figure 4B**) revealed a significant difference in the overall variance of daily salivary cortisol changes between intensity conditions (*t _(48)_* = -2.271, *p* = 0.028, *Cohen’s d* = -0.642). This suggests that negative intensity influences the habituation process of salivary cortisol: high-intensity negative stimuli elicit a greater overall magnitude of habituation compared with moderate-intensity stimuli.

**Figure 4:**
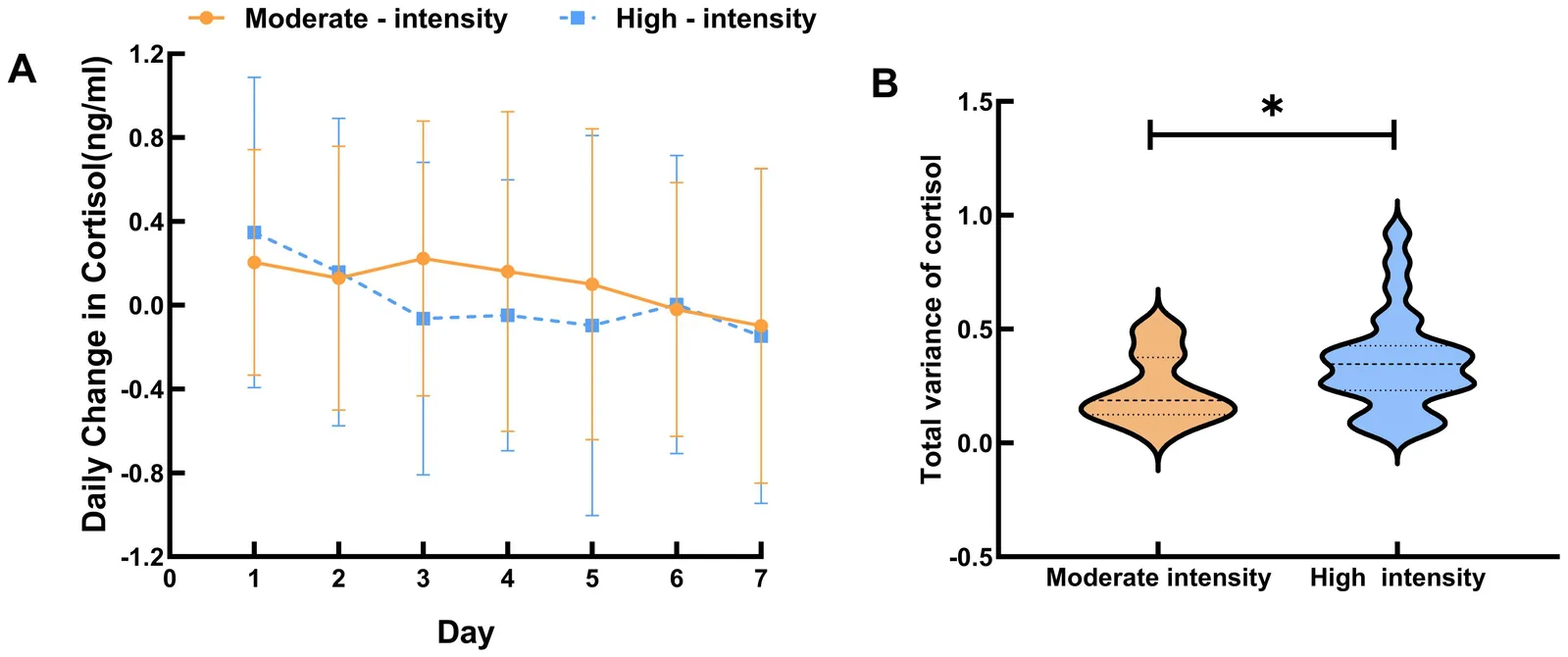
Tests of differences in cortisol. **(A)** Results of daily salivary cortisol changes across seven consecutive experimental days under moderate-intensity and high-intensity conditions. **(B)** Violin plots of data distributions under different negative intensity conditions, using the overall variance of daily cortisol changes across seven days as habituation indices. *p < 0.05.

### 3.4 Correlations among variables under different negative intensities

To further examine the relationships among subjective experience, eye-movement behavior, and HPA-axis activity, we conducted correlation analyses within each negative intensity condition using the 7-day total variance of each variable as the habituation index (see **Table 3**). Results showed that under moderate intensity, valence was positively correlated with arousal (*r* = 0.465, *p* < 0.05) and cortisol (*r* = 0.542, *p* < 0.01), whereas correlations with MFD and pupil size were nonsignificant. Under high intensity, only the correlation between valence and arousal was significant (*r* = 0.599, p < 0.01), and all other pairwise associations were nonsignificant. These results suggest that under moderate intensity, the progression speed of habituation is synchronized between subjective experience and endocrine responses, whereas under high intensity this cross-domain synchrony diminishes.

**Table 3:** Correlations among variables under different negative intensities.

Variable	Moderate intensity					High intensity				
	Valence	Arousal	Cortisol	MFD	Pupil size	Valence	Arousal	Cortisol	MFD	Pupil size
Valence	1					1				
Arousal	0.465*	1				0.599**	1			
Cortisol	0.542**	0.289	1			-0.071	-0.013	1		
MFD	0.053	-0.18	-0.069	1		0.078	0.002	0.09	1	
Pupil size	0.256	0.069	0.051	0.16	1	0.241	0.165	-0.044	0.073	1

MFD, Mean fixation duration; *∗p* < 0.05;**p<0.01.

## 4 Discussion

To the best of our knowledge, this is the first study to examine the physiological and behavioral mechanisms of NEH across three levels—subjective experience, eye-movement behavior, and HPA-axis activity. The findings showed that after multi-day repeated exposure to negative stimuli, subjective valence ratings increased significantly, while arousal ratings decreased significantly. At the attentional level, MFD increased significantly, whereas pupil size decreased significantly. At the physiological level, daily change in salivary cortisol showed a significant downward trend, with greater decreases under high-intensity negative stimulation, suggesting a greater overall magnitude of habituation.

Notably, inter-individual differences did not act uniformly across levels. Psychological resilience exerted a latent moderating effect on the association between trait anxiety and valence habituation at the subjective level. Judging from the marginally significant interaction trend, individuals with high resilience tended to exhibit a greater reduction in negative valence responses compared to those with low resilience. Furthermore, as the level of trait anxiety increased, the overall habituation magnitude declined among high-resilience individuals, whereas the opposite pattern was observed in low-resilience individuals. In addition, trait anxiety was negatively associated with habituation of the pupillary response, such that higher trait anxiety corresponded to slower habituation of pupil size. Taken together, our results indicate that NEH is not a single pathway but a dynamic process jointly accomplished by distinct systems with different sensitivities and convergence rhythms.

### 4.1 Subjective response findings

Our results indicate that resilience potentially moderated the association between trait anxiety and valence habituation. According to diathesis–stress theories (21–23), mental health outcomes are jointly determined by inherent or early-acquired vulnerabilities and later life stressors. This framework emphasizes that vulnerability factors (e.g., trait anxiety) and protective factors (e.g., psychological resilience) jointly shape an individual’s stress–habituation trajectory. High trait anxiety is associated with heightened threat vigilance, negative expectancy bias, and difficulty disengaging attention, whereas resilience reflects rapid recovery, cognitive reappraisal, and resource mobilization after stress; these capacities may stabilize subjective experience and buffer the adverse effects of anxiety (12, 15, 16). Accordingly, in the present study, higher trait anxiety likely heightened sensitivity to threat cues. Among high-resilience individuals, such excessive threat-focused interference may have slowed the course of valence habituation (4). In low-resilience individuals, anxiety-related threat sensitivity likely elevates initial discomfort, which then yields a larger discrepancy across days under repeated, predictable exposure, strengthening inhibitory learning and reappraisal signals and producing more pronounced changes in valence.

Moreover, from alternative viewpoints, the presence of a ceiling effect is also plausible. Specifically, individuals with high resilience may already exhibit very rapid initial adaptation on Day 1, leaving limited room for further reduction in valence reactivity across subsequent days, thus resulting in smaller overall variance. In contrast, low-resilience individuals start with stronger initial responses and show greater observable change across the 7-day period. Nevertheless, this preliminary finding requires replication with a larger sample population.

### 4.2 Attentional processing findings

We found that higher-intensity negative stimuli were associated with longer MFD and larger pupils, indicating stronger attentional bias and emotional arousal. These findings accord with vigilance–avoidance theory and prior work on attentional bias (4, 6, 7, 11). Early in exposure, individuals typically exhibit heightened vigilance, rapidly scanning threat cues with elevated arousal; accordingly, pupils are larger and fixations shorter at the outset. With increasing repetitions, avoidance tendencies wane and attention shifts from defensive monitoring to more stable sustained processing, evidenced by longer mean fixations and reduced physiological arousal (pupil constriction) (7, 10). However, when habituation speed was indexed by the 7-day total variance, no significant difference emerged between intensity conditions. A likely explanation is that stimulus intensity primarily elevates baseline levels, whereas repeated, multi-day, same-procedure exposure increases contextual familiarity and reduces uncertainty.

Notably, we also observed a significant negative association between trait anxiety and pupillary habituation. According to Attentional Control Theory (37), elevated anxiety weakens top-down control while amplifying bottom-up threat-driven processing. Consequently, during repeated exposure, individuals tend to remain in a fixed “vigilance–monitoring” mode, yielding constrained pupillary modulation and reduced variability (38). By contrast, when anxiety is lower, attentional control is more intact, enabling a shift from early defensive orienting to sustained processing and reappraisal, thereby producing faster habituation across repeated exposure. This finding is also consistent with the clinical empirical observation that individuals with high anxiety exhibit “slow improvement and proneness to residual symptoms”, and it provides evidence for incorporating attention control strategies into exposure therapy (39).

### 4.3 Neuroendocrine response findings

According to the inhibitory learning model, the key mechanism of exposure is not “response suppression” but repeatedly experiencing the nonoccurrence of the expected threat, thereby forming new “safe/harmless” learning (40, 41). High-intensity cues elicit stronger threat expectancies on day one; thus, when “no actual harm” repeatedly occurs, the expectancy violation is larger, yielding a stronger safety-learning signal and faster consolidation. Consequently, in our study this manifested as more pronounced declines in cortisol across the first few days under high-intensity exposure. Meanwhile, the allostatic load model provides a physiological account for these findings (42). Early high-intensity exposure evokes higher cortisol peaks and more fully engages glucocorticoid receptor–mediated HPA-axis negative feedback; under predictable, controllable repetition, this feedback is effectively “rehearsed” daily, driving ACTH/HPA output back to lower levels more rapidly, whereas the moderate-intensity response is smaller, with gentler onset and consolidation of feedback, yielding a more gradual decline (43).

We found that only under moderate-intensity negative stimulation was the speed of cortisol habituation associated with valence rather than arousal. This pattern aligns with a controllability appraisal pathway: as individuals increasingly judge the context to be predictable and the outcome controllable, what changes first is the evaluation of the stimulus’s negative meaning (valence) (40, 41). The HPA axis is more sensitive to this reappraisal of controllability; accordingly, cortisol habituation tracks changes in valence rather than fluctuations in arousal (17). In other words, within this paradigm, meaning updates arising from feeling in control better propel endocrine habituation than the immediate intensity of arousal. Clinically, these results suggest using valence ratings and cortisol as relatively concordant process markers during moderate-intensity exposure; when facing high-intensity cues, one should anticipate asynchrony between subjective and physiological responses and consider staged exposure or attention training to promote re-coupling. Of course, these inferences are correlational and warrant confirmation in larger samples and longitudinal causal models.

### 4.4 Limitations and future directions

It must be acknowledged that this study has several limitations. First, all participants were healthy male university students with a relatively modest sample size. The exclusive inclusion of male participants represents an independent limitation on generalizability: given well-documented sex differences in HPA axis reactivity and emotional processing, the current findings cannot be directly generalized to female populations, constraining the external validity of conclusions for the full adult population. Second, this study employed a passive viewing paradigm with an identical image set presented across all seven testing days, and no neutral control condition was included. Under this design, the observed habituation effects may partly reflect stimulus recognition and familiarity rather than pure emotional adaptation per se. Third, cortisol secretion is inherently influenced by diurnal rhythms and daily lifestyle factors. Although standardized control procedures were implemented to minimize such confounds, denser sampling across each testing session would improve the temporal resolution of HPA axis response characterization. Additionally, the primary cortisol metric used in this study differs from conventional HPA habituation measures widely used in the literature.

Building on the current findings and limitations, several directions for future research are proposed. First, future studies should expand the sample size and recruit female participants to systematically examine sex differences in multi-system long-term emotional habituation, thereby improving the external validity of the findings. Second, refinements to the experimental design are recommended. Adding a neutral image control condition and interspersing novel negative stimuli across testing days would help dissociate genuine emotional habituation from general stimulus familiarity and practice effects. Third, combining variance-based habituation indices with Day 1–Day 7 difference scores and other complementary analytical approaches would allow for a more comprehensive characterization of both the full dynamic trajectory and the net magnitude of adaptive change, advancing understanding of long-term emotional habituation processes.

## 5 Conclusions

In summary, our findings indicate that habituation to negative emotion is not a single, synchronous trajectory; rather, under the joint influence of time, stimulus intensity, and individual traits, it unfolds differently across subjective experience, attention, and the HPA axis: time facilitates a decline in subjective distress, higher intensity more readily accelerates stabilization of endocrine responses, and anxiety and resilience selectively shape the form of multi-day trajectories. Incorporating these three classes of indices into a unified, repeatable follow-up framework may enable joint consideration of “feeling better” and “physiological stabilization,” thereby providing a more reliable basis for implementing exposure-based interventions, monitoring progress, and preventing relapse.

## Data Availability

The raw data supporting the conclusions of this article will be made available by the authors, without undue reservation.
