# Supplementary material for: The dynamic features of long-term habituation of negative emotions: evidence from eye-tracking and hypothalamic-pituitary-adrenal axis research
Source: Front Psychiatry. 2026 Oct 7;17:1845849. doi: 10.3389/fpsyt.2026.1845849 (PMC13683772; doi:10.3389/fpsyt.2026.1845849)
Supplement: Supplementary file 1 [file Table1.docx]

| **Table S1. Descriptive Statistics** | | | | | | | | |
| --- | --- | --- | --- | --- | --- | --- | --- | --- |
| **Group** | **Collection Days** | **Subjective Evaluation** | |  | **Eye-Tracking Indicators** | |  | **Cortisol Change** |
|  |  | **Valence** | **Arousal** |  | **Pupil Size** | **Mean Fixation Duration** |  |  |
| **Moderate Intensity**  **（n=25）** | Day 1 | 3.545（1.113） | 4.474（2.084） |  | 72.891（5.245） | 16.754（1.300） |  | 0.204（0.539） |
|  | Day 2 | 3.429（0.735） | 4.249（2.219） |  | 71.671（5.555） | 17.000（0.978） |  | 0.129（0.630） |
|  | Day 3 | 3.464（0.814） | 4.357（2.339） |  | 71.334（5.499） | 17.205（1.229） |  | 0.222（0.656） |
|  | Day 4 | 3.525（0.844） | 4.359（2.286） |  | 71.188（4.916） | 17.052（1.170） |  | 0.160（0.763） |
|  | Day 5 | 3.583（0.723） | 4.257（2.021） |  | 71.180（5.208） | 17.329（1.185） |  | 0.100（0.742） |
|  | Day 6 | 3.669（0.646） | 4.146（2.012） |  | 71.480（5.181） | 17.427（1.582） |  | -0.021（0.606） |
|  | Day 7 | 3.685（0.712） | 4.022（2.011） |  | 71.071（4.991） | 17.632（1.092） |  | -0.098（0.751） |
| **High Intensity**  **（n=25）** | Day 1 | 3.001（0.695） | 4.975（1.701） |  | 75.495（4.746） | 17.309（1.615） |  | 0.347（0.740） |
|  | Day 2 | 3.320（0.833） | 4.531（1.861） |  | 73.973（5.698） | 17.668（1.728） |  | 0.157（0.734） |
|  | Day 3 | 3.375（0.996） | 4.436（2.055） |  | 74.606（4.899） | 17.753（1.659） |  | -0.064（0.745） |
|  | Day 4 | 3.382（1.074） | 4.437（2.194） |  | 74.083（4.902） | 17.672（1.477） |  | -0.049（0.647） |
|  | Day 5 | 3.458（1.059） | 4.242（2.125） |  | 73.304（4.499） | 17.916（1.506） |  | -0.097（0.907） |
|  | Day 6 | 3.485（1.068） | 4.250（2.192） |  | 74.147（4.849） | 18.269（1.551） |  | 0.003（0.711） |
|  | Day 7 | 3.529（1.068） | 4.151（2.209） |  | 73.553（4.518） | 18.749（1.498） |  | -0.148（0.798） |

Note. Values in the table are presented as Mean (SD); Eye-Tracking Indicators are values after square root transformation; Cortisol Change is calculated as (post-experiment concentration - pre-experiment concentration) per day, with values converted by logarithm, and the unit is ng/ml.

| **Table S2. Post-hoc multiple comparisons of the main effect of time on subjective ratings** | | | | | | | |
| --- | --- | --- | --- | --- | --- | --- | --- |
| **Dimension** | **Time** | **Time** | **Mean** | **SD** | **p** | **95% CI** | |
|  |  |  |  |  |  | **LL** | **UL** |
| **Valence** | 1 | 2 | -0.101 | 0.113 | 0.375 | -0.328 | 0.126 |
|  |  | 3 | -0.146 | 0.121 | 0.232 | -0.389 | 0.097 |
|  |  | 4 | -0.180 | 0.13 | 0.172 | -0.442 | 0.081 |
|  |  | 5 | -0.247 | 0.138 | 0.079 | -0.525 | 0.030 |
|  |  | 6 | -0.304* | 0.136 | 0.030 | -0.577 | -0.030 |
|  |  | 7 | -0.333* | 0.141 | 0.022 | -0.616 | -0.051 |
|  | 2 | 3 | -0.045 | 0.043 | 0.302 | -0.132 | 0.042 |
|  |  | 4 | -0.079 | 0.055 | 0.158 | -0.19 | 0.032 |
|  |  | 5 | -0.146* | 0.069 | 0.039 | -0.284 | -0.008 |
|  |  | 6 | -0.203* | 0.078 | 0.012 | -0.359 | -0.046 |
|  |  | 7 | -0.232* | 0.084 | 0.008 | -0.402 | -0.062 |
|  | 3 | 4 | -0.034 | 0.031 | 0.278 | -0.096 | 0.028 |
|  |  | 5 | -0.101 | 0.055 | 0.073 | -0.212 | 0.010 |
|  |  | 6 | -0.158* | 0.069 | 0.028 | -0.297 | -0.018 |
|  |  | 7 | -0.187* | 0.073 | 0.014 | -0.334 | -0.040 |
|  | 4 | 5 | -0.067 | 0.052 | 0.206 | -0.172 | 0.038 |
|  |  | 6 | -0.124 | 0.065 | 0.064 | -0.254 | 0.007 |
|  |  | 7 | -0.153* | 0.070 | 0.034 | -0.295 | -0.012 |
|  | 5 | 6 | -0.057 | 0.031 | 0.071 | -0.118 | 0.005 |
|  |  | 7 | -0.086* | 0.037 | 0.023 | -0.160 | -0.012 |
|  | 6 | 7 | -0.030 | 0.025 | 0.239 | -0.080 | 0.020 |
| **Arousal** | 1 | 2 | 0.335* | 0.117 | 0.006 | 0.100 | 0.569 |
|  |  | 3 | 0.328* | 0.126 | 0.012 | 0.075 | 0.581 |
|  |  | 4 | 0.327* | 0.132 | 0.017 | 0.062 | 0.591 |
|  |  | 5 | 0.476* | 0.160 | 0.005 | 0.153 | 0.798 |
|  |  | 6 | 0.527* | 0.18 | 0.005 | 0.166 | 0.888 |
|  |  | 7 | 0.638* | 0.194 | 0.002 | 0.249 | 1.027 |
|  | 2 | 3 | -0.007 | 0.070 | 0.924 | -0.147 | 0.134 |
|  |  | 4 | -0.008 | 0.083 | 0.924 | -0.175 | 0.159 |
|  |  | 5 | 0.141 | 0.122 | 0.255 | -0.105 | 0.386 |
|  |  | 6 | 0.192 | 0.148 | 0.200 | -0.105 | 0.490 |
|  |  | 7 | 0.303 | 0.164 | 0.071 | -0.027 | 0.634 |
|  | 3 | 4 | -0.001 | 0.047 | 0.979 | -0.095 | 0.093 |
|  |  | 5 | 0.147 | 0.102 | 0.156 | -0.058 | 0.353 |
|  |  | 6 | 0.199 | 0.129 | 0.129 | -0.060 | 0.458 |
|  |  | 7 | .310* | 0.149 | 0.043 | 0.010 | 0.610 |
|  | 4 | 5 | 0.149 | 0.091 | 0.108 | -0.034 | 0.331 |
|  |  | 6 | 0.200 | 0.116 | 0.091 | -0.033 | 0.434 |
|  |  | 7 | 0.311* | 0.138 | 0.029 | 0.034 | 0.589 |
|  | 5 | 6 | 0.051 | 0.046 | 0.268 | -0.041 | 0.144 |
|  |  | 7 | 0.163* | 0.068 | 0.020 | 0.026 | 0.299 |
|  | 6 | 7 | 0.111* | 0.046 | 0.019 | 0.019 | 0.203 |

Note. 95% CI：95% Confidence Interval for the Difference； LL： Lower Limit； UL: Upper Limit. *p<0.05. Multiple comparison: Least Significant Difference (LSD) method.

| **Table S3. Post-hoc multiple comparisons of the main effect of time on eye-tracking indicators.** | | | | | | | |
| --- | --- | --- | --- | --- | --- | --- | --- |
| **Dimension** | **Time** | **Time** | **Mean** | **SD** | **p** | **95% CI** | |
|  |  |  |  |  |  | **LL** | **UL** |
| **Mean fixation duration** | 1 | 2 | -0.301 | 0.153 | 0.054 | -0.609 | 0.006 |
|  |  | 3 | -0.448* | 0.188 | 0.021 | -0.826 | -0.069 |
|  |  | 4 | -0.330* | 0.151 | 0.033 | -0.633 | -0.027 |
|  |  | 5 | -0.591* | 0.211 | 0.007 | -1.015 | -0.167 |
|  |  | 6 | -0.817* | 0.216 | 0.001 | -1.251 | -0.383 |
|  |  | 7 | -1.159* | 0.198 | 0.001 | -1.557 | -0.762 |
|  | 2 | 3 | -0.146 | 0.140 | 0.301 | -0.427 | 0.135 |
|  |  | 4 | -0.029 | 0.141 | 0.839 | -0.313 | 0.256 |
|  |  | 5 | -0.289 | 0.165 | 0.086 | -0.621 | 0.043 |
|  |  | 6 | -0.515* | 0.172 | 0.004 | -0.860 | -0.170 |
|  |  | 7 | -0.858* | 0.196 | 0.001 | -1.252 | -0.464 |
|  | 3 | 4 | 0.117 | 0.155 | 0.453 | -0.195 | 0.429 |
|  |  | 5 | -0.143 | 0.127 | 0.264 | -0.398 | 0.112 |
|  |  | 6 | -0.369* | 0.161 | 0.026 | -0.693 | -0.046 |
|  |  | 7 | -0.712* | 0.181 | 0.001 | -1.075 | -0.349 |
|  | 4 | 5 | -0.26 | 0.158 | 0.105 | -0.578 | 0.057 |
|  |  | 6 | -0.487* | 0.153 | 0.003 | -0.794 | -0.179 |
|  |  | 7 | -0.829* | 0.173 | 0.001 | -1.176 | -0.482 |
|  | 5 | 6 | -0.226 | 0.150 | 0.138 | -0.527 | 0.075 |
|  |  | 7 | -0.569* | 0.149 | 0.001 | -0.868 | -0.269 |
|  | 6 | 7 | -0.343* | 0.163 | 0.041 | -0.670 | -0.015 |
| **Pupil size** | 1 | 2 | 1.371* | 0.381 | 0.001 | 0.604 | 2.138 |
|  |  | 3 | 1.223* | 0.382 | 0.002 | 0.455 | 1.991 |
|  |  | 4 | 1.557* | 0.458 | 0.001 | 0.637 | 2.477 |
|  |  | 5 | 1.951* | 0.462 | 0.001 | 1.023 | 2.880 |
|  |  | 6 | 1.380* | 0.528 | 0.012 | 0.319 | 2.441 |
|  |  | 7 | 1.881* | 0.503 | 0.001 | 0.871 | 2.891 |
|  | 2 | 3 | -0.148 | 0.302 | 0.627 | -0.756 | 0.460 |
|  |  | 4 | 0.186 | 0.417 | 0.657 | -0.652 | 1.024 |
|  |  | 5 | 0.580 | 0.400 | 0.153 | -0.224 | 1.385 |
|  |  | 6 | 0.009 | 0.472 | 0.985 | -0.939 | 0.957 |
|  |  | 7 | 0.510 | 0.441 | 0.253 | -0.377 | 1.397 |
|  | 3 | 4 | 0.334 | 0.295 | 0.263 | -0.259 | 0.927 |
|  |  | 5 | 0.728* | 0.311 | 0.023 | 0.104 | 1.353 |
|  |  | 6 | 0.157 | 0.399 | 0.696 | -0.646 | 0.959 |
|  |  | 7 | 0.658 | 0.387 | 0.096 | -0.120 | 1.437 |
|  | 4 | 5 | 0.394 | 0.317 | 0.219 | -0.243 | 1.031 |
|  |  | 6 | -0.177 | 0.371 | 0.635 | -0.924 | 0.569 |
|  |  | 7 | 0.324 | 0.321 | 0.318 | -0.322 | 0.970 |
|  | 5 | 6 | -0.572* | 0.274 | 0.042 | -1.122 | -0.021 |
|  |  | 7 | -0.070 | 0.270 | 0.796 | -0.614 | 0.473 |
|  | 6 | 7 | 0.501 | 0.314 | 0.117 | -0.130 | 1.133 |

Note. 95% CI：95% Confidence Interval for the Difference； LL： Lower Limit； UL: Upper Limit. *p<0.05. Multiple comparison: Least Significant Difference (LSD) method.

| **Table S4. Post-hoc multiple comparisons of the main effect of time on cortisol change.** | | | | | | |
| --- | --- | --- | --- | --- | --- | --- |
| **Time** | **Time** | **Mean** | **SD** | **p** | **95% CI** | |
|  |  |  |  |  | **LL** | **UL** |
| 1 | 2 | 0.132 | 0.111 | 0.239 | -0.091 | 0.356 |
|  | 3 | 0.196 | 0.122 | 0.113 | -0.048 | 0.441 |
|  | 4 | 0.22 | 0.129 | 0.096 | -0.04 | 0.48 |
|  | 5 | 0.274 | 0.137 | 0.052 | -0.002 | 0.55 |
|  | 6 | .284* | 0.121 | 0.023 | 0.042 | 0.527 |
|  | 7 | .398* | 0.14 | 0.007 | 0.116 | 0.681 |
| 2 | 3 | 0.064 | 0.113 | 0.574 | -0.163 | 0.29 |
|  | 4 | 0.087 | 0.12 | 0.473 | -0.155 | 0.329 |
|  | 5 | 0.142 | 0.146 | 0.339 | -0.153 | 0.436 |
|  | 6 | 0.152 | 0.11 | 0.175 | -0.07 | 0.374 |
|  | 7 | .266* | 0.107 | 0.016 | 0.051 | 0.48 |
| 3 | 4 | 0.023 | 0.123 | 0.849 | -0.223 | 0.27 |
|  | 5 | 0.078 | 0.147 | 0.6 | -0.219 | 0.374 |
|  | 6 | 0.088 | 0.114 | 0.446 | -0.142 | 0.318 |
|  | 7 | 0.202 | 0.125 | 0.114 | -0.05 | 0.454 |
| 4 | 5 | 0.054 | 0.106 | 0.612 | -0.16 | 0.268 |
|  | 6 | 0.065 | 0.123 | 0.601 | -0.182 | 0.311 |
|  | 7 | 0.179 | 0.114 | 0.123 | -0.05 | 0.407 |
| 5 | 6 | 0.01 | 0.142 | 0.943 | -0.274 | 0.295 |
|  | 7 | 0.124 | 0.125 | 0.325 | -0.127 | 0.375 |
| 6 | 7 | 0.114 | 0.126 | 0.371 | -0.14 | 0.368 |

Note. 95% CI：95% Confidence Interval for the Difference； LL： Lower Limit； UL: Upper Limit. *p<0.05. Multiple comparison: Least Significant Difference (LSD) method.
